# Complete Genome Sequence of the Avian-Pathogenic *Escherichia coli* Strain APEC O18

**DOI:** 10.1128/genomeA.01213-16

**Published:** 2016-11-03

**Authors:** Bryon A. Nicholson, Yvonne M. Wannemuehler, Catherine M. Logue, Ganwu Li, Lisa K. Nolan

**Affiliations:** a Department of Veterinary Microbiology and Preventive Medicine, College of Veterinary Medicine, VMRI 2, Iowa State University, Ames, Iowa, USA; b Department of Veterinary Diagnostic and Production Animal Medicine, College of Veterinary Medicine, Iowa State University, Ames, Iowa, USA

## Abstract

Avian-pathogenic *Escherichia coli* (APEC) is the causative agent of colibacillosis, a disease that affects all facets of poultry production worldwide, resulting in multimillion dollar losses annually. Here, we report the genome sequence of an APEC O18 sequence type 95 (ST95) strain associated with disease in a chicken.

## GENOME ANNOUNCEMENT

Extraintestinal pathogenic *Escherichia coli* (ExPEC) strains are responsible for some of the most widespread and consequential diseases of human and animal hosts. Genetic similarities between human and avian ExPEC strains plus similarities in the mechanisms by which they cause disease have led to the suspicion that some ExPEC are zoonotic pathogens. Especially notable in this regard are neonatal meningitis *E. coli* (NMEC) and avian-pathogenic *E. coli* (APEC). Here, we document the first fully closed genomic sequence of an avian-pathogenic *E. coli* strain of serogroup O18. APEC O18 shows potential as a zoonotic pathogenic capable of causing neonatal meningitis in human newborns.

APEC O18 is a serogroup O18 sequence type 95 (ST95) strain isolated from the pericardial and lung tissue of a case of colibacillosis in Nebraska, isolated prior to 2000. APEC O18, previously labeled APEC 380, has been characterized in the chicken model of colibacillosis, where it caused 80% mortality at 24 h, and in the rat model of neonatal meningitis, where it causes 75% mortality at 24 h (1).

Genomic sequencing was performed using complementary sequencing technologies, combining results obtained from a Roche/454 FLX genome sequencer (GS) instrument and an Illumina HiSeq 2000. The following data sets were used in the final assembly: (i) GS-FLX, with 152,602 paired reads and 423,865 shotgun reads totaling 155 Mb (~31-fold coverage), and (ii) Illumina 100-bp paired-end library with 11,193,121 reads totaling 839 Mb (~168-fold coverage). Both 454-read sets were assembled *de novo* using Newbler 2.6 (Roche), and Illumina reads were assembled separately with Velvet 1.1 (2). The genome was closed using 454 assemblies as a reference sequence with the Illumina data used to add depth, correct errors, and close gaps. Whole-genome optical mapping (OpGen, Gaithersburg, MD) was used to validate scaffolds and contig order. The assembly was confirmed using PCR and Sanger sequencing and validated by the consistency of paired-end evidence from 454 and Illumina reads.

The assembled genome consists of a single chromosome (5,006,568 bp; 50.70% G+C content) containing 4,674 protein-coding genes and 85 tRNA-carrying genes. The APEC O18 is larger than previously sequenced strains APEC O78 (3) and APEC χ7122 (GenBank accession no. CAJR00000000.1) and shares similarities to strain APEC O1 (4), a strain which does not cause disease in the meningitis model. Genomic comparisons of this isolate with other sequenced ExPEC genomes is ongoing.

### Accession number(s).

The whole-genome sequence of APEC O18 has been deposited in GenBank under the accession number CP006830.
